# Abcès thyroïdien à Escherichia coli: à propos d’un cas et revue de la littérature

**Published:** 2012-03-09

**Authors:** Malika Fassih, Essadik Moujahid, Reda Abada, Sami Rouadi, Mohamed Mahtar, Mohamed Roubal, Moustapha Essadi, Mohamed Fatmi El Kadiri

**Affiliations:** 1 Service d’ORL et de Chirurgie cervico-faciale, Hôpital 20 Août, CHU Ibn Rochd - Casablanca, Maroc

**Keywords:** Glande thyroïde, abcès de la thyroïde, thyroïdite, goitre, infection, Maroc

## Abstract

L’abcès de la thyroïde est une entité pathologique extrêmement rare, représentant seulement 0.1% des pathologies chirurgicales de la thyroïde. C’est une situation inhabituelle en raison des caractéristiques anatomiques et physiologiques de la glande qui lui donne une capacité de résistance vis-à-vis des infections. Nous rapportons un cas rare d’abcès thyroïdien à Escherichia. coli survenu chez une patiente de 55 ans, immunodéprimée: diabétique insulinodépendante non équilibrée, hypertendue mal suivie avec insuffisance cardiaque gauche, et en insuffisance rénale chronique terminale. La patiente s’est présentée aux urgences avec une tuméfaction basi-cervicale antérieure évoluant sur 10 jours, prédominante à droite, mesurant 6 cm, sensible, inflammatoire, mobile à la déglutition, avec une dyspnée mixte, sueurs et fièvre. Un scanner cervico-thoracique mettait en évidence un énorme processus du lobe thyroïdien droit à contenu liquidien de 9cm, refoulement des voies aériennes supérieures. Une cytoponction à l’aiguille ramenait 10 ml de liquide purulent. Le drainage chirurgical a été réalisé sous anesthésie locale vue le risque d’intubation difficile devant le blindage cervical en présence d’une trachée difficilement accessible. Une E. coli était isolée après la culture du liquide de drainage. La patiente était porteuse d’un bricker depuis l’enfance pour malformation de la vessie. Ceci suggère une bactériémie à point de départ urinaire, avec embole septique au niveau de la glande thyroïde. L’évolution était bonne sous tri-antibiothérapie, et des pansements biquotidiens, avec équilibration de son diabète et insuffisance rénale chronique. Nous rappellerons à travers cette observation les moyens de défense de la thyroïde contre les infections, les différents facteurs prédisposant à l’abcès de la thyroïde, et les germes incriminés dans chaque étiologie. Nous discuterons les diagnostics différentiels et nous insisterons sur les modalités diagnostiques et de prise en charge thérapeutique de ce type de malades.

## Introduction

L’abcès de la thyroïde est une entité pathologique rarement documentée dans la littérature, représentant seulement 0.1% des pathologies chirurgicales de la thyroïde. Le caractère inhabituel est attribué aux caractéristiques anatomique et physiologique de la glande thyroïde qui lui confère une résistance unique vis à vis des infections. Ils surviennent généralement sur une glande de morphologie anormale acquise (goitre, nodule), anomalie congénitale (fistule de la 4ème fente), ou iatrogène (cytoponction). Chez l’enfant, il est souvent associé à une fistule au niveau du sinus piriforme, toutefois, la cause chez l’adulte est souvent une dissémination à partir d’un foyer infectieux de proximité ou à distance (bactériémie). L’association à un goitre ancien, ou une néoplasie thyroïdienne, est un facteur prédisposant.

Si les infections à Streptococcus sp et Staphylococcus sp sont les plus fréquentes, l′implication d’autres agents pathogènes (agents fongiques, Bacilles Gram Négatif, germes anaérobies) est rarement rapportée dans la littérature, et souvent en rapport avec une immunodépression.

Les thyroïdites virales et chroniques étant les plus rencontrées en pratique, Le diagnostic de l’abcès thyroïdien est souvent fait tardivement ce qui prédispose à de graves complications altérant le pronostic fonctionnel, par lésion des structures voisines, et vital.

Nous rapportant le cas d’une patiente de 55 ans, avec un abcès de la thyroïde, sur terrain multi-taré, diagnostiquée tardivement au stade de complications: compression de la filière aérienne, thrombophlébite de la veine jugulaire interne. L’isolement de *Escherichia coli* dans le liquide de drainage a permis de conclure à une bactériémie à point de départ urinaire, l’hypothèse est confortée par les antécédents de la patiente porteuse d’une malformation urinaire, et les résultats de l’examen cytobactériologique des urine (ECBU).

## Patient et observation

Nous rapportons un cas rare d′abcès thyroïdien à *Escherichia coli* survenu chez une patiente de 55 ans, immunodéprimée: diabétique insulinodépendante non équilibrée, hypertendue mal suivie avec insuffisance cardiaque gauche, et en insuffisance rénale chronique terminale. Elle n’avait aucun antécédent de pathologie thyroïdienne sous-jacente. La patiente s’est présentée aux urgences avec une tuméfaction basi-cervicale antérieure évoluant sur 10 jours. Les premiers symptômes étaient: gène à la déglutition, asthénie et une difficulté à respirer mise sur le compte de son insuffisance cardiaque. La symptomatologie s’est aggravée au fil des jours, avec apparition d’une tuméfaction cervicale antérieure augmentant progressivement de taille, associée à une dyspnée aggravée au décubitus, une aphagie, dans un contexte d ‘altération de l’état général et de fièvre.

**Figure 1:**
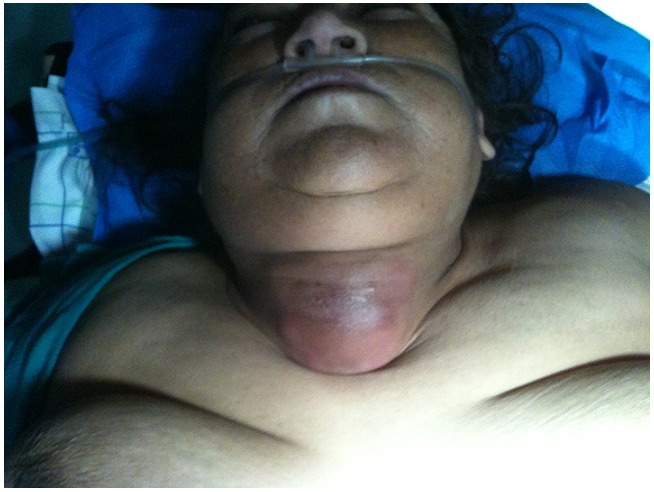
Abcès de la thyroïde: masse basicervicale, médiane, inflammatoire, et tendue

**Figure 2:**
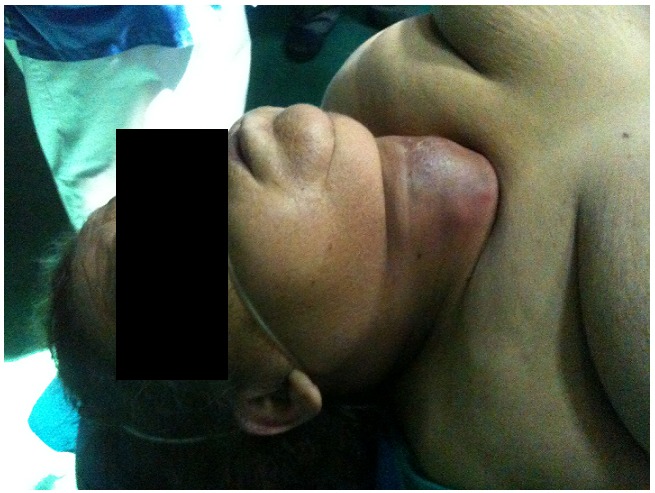
Abcès thyroïdien vu de profil, élargissement du diamètre cervical antéropostérieur, témoignant du degré de compression trachéale

L’examen physique a trouvé une masse cervicale basse médiane, prédominante à droite, mesurant 6 cm de grand axe, tendue et rénitente, très douloureuse à la palpation, inflammatoire, mobile à la déglutition, avec une dyspnée mixte, sueurs et fièvre chiffrée à 39°C. (Figure 1 et Figure 2) Un bilan biologique réalisé en premier a montré des perturbations biologiques importantes: La glycémie à jeun était à 3,9g/l avec de l’acétone dans les urines témoignant d’une cétose diabétique. La glycémie à jeun était à 6,26g/l avec de l’acétone dans les urines témoignant d’une cétose diabétique. L’urée était à 1,18 g/l, la créatininémie à 33mg/l, la kaliémie était élevée à 7,04mmol/l, le sodium à 132mmol/l.

L’hémograrmme a objectivé une anémie à 7,8g/dL, une hyperleucocytose à 26,3x10^3^/uL. Un prélèvement pour dosage de la TSHµs et des hormones thyroïdiennes a été effectué. La correction des troubles étant entreprise en urgence en milieu de réanimation, un scanner cervico-thoracique, a été demandé en urgence, objectivant un énorme processus du lobe thyroïdien droit à contenu liquidien de 9cm, avec effet de masse sur les voies aérodigestifs supérieures, et refoulant l’axe jugulo-carotidien droit latéralement. Une thrombose de la veine jugulaire droite a été mise en évidence. L’infiltration des parties molles pré vertébrales et cervicales était importante.

Le diagnostic d’abcès de la thyroïde était évoqué, à côté d’une tumeur thyroïdienne nécrosée. La cytoponction à l′aiguille a ramenée 10 ml de liquide purulent jaunâtre a permis de confirmer le diagnostic d’abcès thyroidien.

Le drainage chirurgical a été décidé. L’intervention a été réalisé sous anesthésie locale vue le risque d’intubation difficile devant le blindage cervical et en présence d’une trachée profonde difficilement accessible (trachéotomie difficile). Le drainage a permis d’évacuer 100 ml de pus. L’amélioration de la dyspnée était précoce dès la fin de l’intervention.

*E. coli* a été isolée après la culture du liquide de drainage. La patiente était porteuse d’un bricker depuis l’enfance pour une malformation de la vessie (non documentée). Ceci suggère une bactériémie à point de départ urinaire, avec embole septique au niveau de la glande thyroïde. Un ECBU réalisé après avoir démarré l’antibiothérapie probabiliste est revenu stérile, mais avec la présence d’un nombre significativement élevé de leucocytes altérées témoignant d’une infection en cours. L’hémoculture est revenue stérile. Le bilan hormonal était normal.

L’évolution était bonne sous tri-antibiothérapie première à base de céftriaxone 2 g/j+ flgyl 2g/j +gentamycine 160mg/j, secondairement adaptée à l’antibiogramme: le germe était sensible à la céftriaxone et à la gentamycine. Des pansements biquotidiens ont été réalisée pendant 2 semaines en hospitalisation et 1 semaine en ambulatoire jusqu’à tarissement de l’infection. L’équilibration du diabète et des troubles hydro-éléctrolytiques n’a été obtenue qu’après 7 jours du drainage chirurgical.

## Discussion

L’abcès de la thyroïde est une entité pathologique non commune, rarement rencontrée en pratique médicale. Elle représente seulement 0,1% des pathologies chirurgicales de la glande thyroïde [1]. En effet, dans une revue de littérature réalisée en 1981, Schweitzer and Olson ont noté que seulement 39 cas d’abcès de la thyroïde ont été rapportés depuis 1951[1]. L’infection de la glande thyroïde est rare, ceci est attribuée en grande partie à sa situation anatomique isolée grâce a sa capsule fibreuse. En outre, elle est dotée d’une vascularisation riche et largement anastomosées, un drainage lymphatique important, et d’une haute concentration en iode. Ce qui lui procure une grande capacité de résistance aux agents pathogènes [2].

L’abcès de la thyroïde touche l’enfant plus que l’adulte. Schweitzer a constaté que parmi les 39 cas d’abcès, 16 étaient des enfants [1]. Ceci est attribué à l’existence de malformations congénitales telles les fistules du tractus thyréoglosse, et les fistules de la 4ème poche endobrachiale qui sont dues à la persistance anormale d’un canal unissant le fond du sinus piriforme à la face profonde d’un lobe thyroïdien. Les patients porteurs de cette anomalie embryologique ont souvent des épisodes récurrents d’abcès cervical bas souvent du côté gauche et ne sont diagnostiqué qu’après opacification des voies digestives hautes montrant le trajet fistuleux [3].

Dans la population adulte, multiple étiologies sont en cause, Une infection peut résulter d’une effraction direct par un corps étranger, tels que la cytoponction thyroïdienne, une perforation de l’œsophage ou de l’hypo pharynx secondaire à l’ingestion d’un os de poulet par exemple, cependant, la dissémination sanguine ou lymphatique à partir d’un foyer à distance est considéré comme la cause la plus commune, la source étant le plus souvent inconnue. L’inoculation est possible par les injections intraveineuses dans le cadre de consommation de drogue. L’extension de l’infection peut se faire à partir des tissus cervicaux contigus, ou d’une fistule du tractus thyréoglosse [4,5].

Une large étude de Yu et al [6], a noté une répartition égale entre les deux sexes. L’abcès de la thyroïde peut survenir à tout âge, la même étude a démontré que cette pathologie peut toucher une large population, à partir de 16 mois à 77 ans.

La fréquence de cette entité est élevée chez la population avec une immunodéficience, telles que l’infection rétrovirale par le VIH, les patients recevant une chimiothérapie, ou des corticoïdes et les sujets transplantés. Notre patiente était diabétique non équilibrée, en insuffisance rénale chronique terminale, ce qui représentent des facteurs importants d’immunodépression [7].

Par ailleurs, Plus des 2/3 des femmes et de la moitié des hommes avec thyroïdites purulentes aigues ont des pathologies thyroïdiennes préexistantes [8] en effet, dans une étude menée au Nigeria, l’incidence des abcès thyroïdiens développés sur un goitre endémique multi-hétéronodulaires était de 10.7% (soit 9 des 84 cas)[9]. Notre patiente n’avait aucun antécédent de pathologies thyroïdiennes sous-jacentes.

Dans une revue de la littérature de l’année 1900 à 1980, la majorité des 224 cas de thyroïdite suppurées qui ont été rapporté, étaient des enfants avec des facteurs prédisposant tels une fistule de la 4ème fente ou fistule du tractus thyréoglosse. L’existence d’une pathologie thyroïdienne, goitre ancien, ou néoplasie thyroïdienne, est reconnu étant un facteur prédisposant chez les adultes [6].

Sur le plan bactériologique, les germes les plus incriminés sont le Staphylococcus aureus, le Streptococcus, et les anaérobies. Ces germes sont retrouvés dans 70% des cas [10]. D’autres espèces ont été isolées: *Escherichia coli* suite à une bactériémie à point de départ urinaire ou digestif [10], *Bacteroides fragilis* isolé chez des femmes en post-hystérectomie [11]. Par ailleurs, d’autres agents pathogènes ont été isolés et rapportés dans la littérature: *Klebsiella*, *Salmonella typhi*, *Acinetobacter*, *Mycobacterium tuberculosis*, *Pseudomonas*, *Eikenella corrodens*, *Clostridium*, *Fusobacterium mortiferum*, *Pneumocystis carnii*, *Haemophilus* [12,7] ainsi que des agents fongiques tels que: *Candida albicans*, et *aspergillosis*[7,13]. Ces espèces ont été identifiées chez des patients avec une immunodépression [14,15].

Cliniquement, l’abcès thyroïdien se présente sous forme d’une tuméfaction cervicale douloureuse faisant suite souvent à une infection respiratoire haute ou pharyngée. Les signes associés sont représentés par l’apparition d’une dyspnée, un enrouement, voire dysphonie, une dysphagie, et une fièvre [3]. Des présentations rares ont été rapportées: paralysie d’une corde vocale, masse cervicale pulsatile, ou des cas asymptomatiques [3,7].

Le diagnostic est confirmé par la ponction à l’aiguille qui ramène du pus franc. L’étude cytobactériologique permet d’isoler l’agent microbien causal, et étudier sa sensibilité aux antibiotiques [3].

Le bilan biologique est souvent perturbé: augmentation de la CRP, hyperleucocytose. Le bilan hormonal est souvent normal, parfois il montre une hyperthyroïdie [2].

L’échographie et le scanner sont d’une aide incontournable dans l’étude de la structure de l’abcès, le nombre de logettes, sa taille, et ses rapports avec les structures anatomiques adjacentes notamment avec paquet vasculo-nerveux du cou et les voies aériennes supérieures [3,16]. Après résolution de l’épisode aigue, le scanner est utile pour rechercher des malformations congénitales tel un trajet fistuleux s’ouvrant dans le sinus piriforme [3,5,16], après ingestion d’un produit hydrosoluble, cette investigation est indispensable spécialement chez les jeunes patients et ceux avec des épisodes récurrents d’abcès thyroïdien [5,7]. Malgré les détails fournis par l’imagerie, il est universellement reconnu que la simple ponction à l’aiguille confirme le diagnostic d’abcès, permettant d’entreprendre un traitement adapté [7].

Les radiographies standards peuvent montrer une déviation de la trachée cervicale [5]. Les diagnostics différentiels sont représentés par les thyroïdites subaigües virales et les thyroïdites chroniques, les hémorragies intra kystiques, les néoplasies primitives ou les métastases, et l’amylose [16].

Dans les thyroïdites d’origine virales, il existe toujours des manifestations systémiques d’hyperthyroïdies orientant le diagnostic. La stratégie thérapeutique repose sur une antibiothérapie probabiliste première adaptée secondairement aux résultats de l’antibiogramme.

L’incision drainage est indiquée à chaque fois qu’il y a une collection purulente objectivée à la ponction à l’aiguille. Sans attendre les signes de souffrance des structures voisines: nerf récurrent, œsophage, larynx…, certains auteurs préconisent une exérèse du lobe thyroïdien siège de l’abcès, ou au minimum un débridement et excision des tissus nécrosés, avec résection des connections fistuleuses si possible [7].

Non traité, l’abcès de la thyroïde peut avoir des conséquences fâcheuses sur les organes voisins. Il peut résulter la destruction du parenchyme glandulaire thyroïdien et des parathyroïdes, une thrombophlébite de la veine jugulaire [5]. Une fistulisation de l’abcès dans l’œsophage ou dans la lumière trachée, fistulisation externe vers la peau [7]. Une septicémie et dissémination sanguine vers des organes à distance [5,7]: ostéomyélite par exemple.

## Conclusion

L’abcès thyroïdien est une entité rare. Le diagnostic est souvent fait tardivement vu le début insidieux et le caractère non spécifique des symptômes. Souvent prise pour thyroïdite subaigüe d’origine virale, l’administration de corticoïdes, traitement de base de cette pathologie, est susceptible d’aggraver le tableau clinique et de disséminer l’infection. C’est une pathologie rapidement progressive dotée d’une morbidité importante, pouvant compromettre le fonctionnement des structures anatomiques adjacentes par compression et nécrose tissulaire, et à distance par dissémination de l’infection. Ces complications peuvent mettre en jeu le pronostic vital. Le diagnostic rapide permettant d’entreprendre un traitement antibiotique précoce et un drainage chirurgical est le seul garant pour prévenir les complications. Le traitement ne doit pas omettre la cure chirurgicale des anomalies congénitales causales: fistule de la 4ème fente, fistule du tractus thyréoglosse, ou des facteurs prédisposant: goitre multinodulaire, ainsi que la correction d’une immunodépression.

## References

[1] Schweitzer VG, Olson NR (1981). Thyroid abscess. Otolaryngol Head Neck Surg..

[2] Berger SA, Zonszein J, Villamena P, Mittman N (1983). Infectious disease of the have defects in host resistance due to diabetes mellitus, cirrhosis, thyroid gland. Rev Infect Dis..

[3] Gan Y, Lam SL (2004). Imaging findings in acute neck infection due to pyriform sinus fistula. Ann Acad Med Singapore..

[4] Deshmukh HG, Verma A, Siegel LB (1994). Stridor, the presenting symptom of a thyroid abscess. Postgrad Med J..

[5] Jacobs A, Gros DA, Gradon JD (2003). Thyroid abscess due to Acinetobacter calcoaceticus: case report and review of the causes of and current management strategies for thyroid abscesses. South Med J..

[6] Yu EH, Ko WC, Chuang YC (1998). Suppurative Acinetobacter baumanii thyroiditis with bacteremic pneumonia: case report and review. Clin Infect Dis..

[7] Herndon MD, Christie DB, Ayoub MM, Duggan AD (2007). Thyroid abscess: case report and review of the literature. Am Surg..

[8] Farwell AP, Braverman LE (1996). Inflammatory thyroid disorders. Otolaryngol Clin North Am..

[9] Ameh EA, Sabo SY, Nmadu PT (1998). The risk of infective thyroiditis in nodular goiters. East Afr Med J..

[10] Sicilia V, Mezitis S (2006). A case of acute suppurative thyroiditis complicated by thyrotoxicosis. J Endocrinol Invest..

[11] Yeluri S, Mehta JP, Karanth S, Dadayal G (2006). A tender lump in the neck. Med J Aust..

[12] Reuben F, Sousa De, Dilip Amonkar, Mervyn Correia (2008). Thyroid Abscess with Cutaneous Fistula: Case Report and Review of the Literature. Thyroid Science..

[13] Lisbona R, Lacourciere Y, Rosenthall L (1973). Aspergillomatous abscesses of the brain and thyroid. J Nucl Med..

[14] Basílio-De-Oliveira CA (2000). Infectious and neoplastic disorders of the thyroid in AIDS patients: an autopsy study. Braz J Infect Dis..

[15] Wang YC, Yeh TS, Lin JD (1997). Gram-negative thyroid abscess resulting from fine-needle aspiration in an immunosuppressed patient. Clin Infect Dis..

[16] Boyce G, Satasivam P, Miller F (2009). Multinodular goitre complicated by abscess due to E. coli. ANZ J Surg..

